# Mortality in Central Java: results from the indonesian mortality registration system strengthening project

**DOI:** 10.1186/1756-0500-3-325

**Published:** 2010-12-02

**Authors:** Chalapati Rao, Soeharsono Soemantri, Sarimawar Djaja, Suhardi, Timothy Adair, Yuana Wiryawan, Lamria Pangaribuan, Joko Irianto, Soewarta Kosen, Alan D Lopez

**Affiliations:** 1 School of Population Health, University of Queensland, 288, Herston Road, Herston, QLD 4006 Australia; 2 National Institute of Health Research and Development, Ministry of Health, 29, Jl. Percetakan Negara, Jakarta 10012, Republic of Indonesia

## Abstract

**Background:**

Mortality statistics from death registration systems are essential for health policy and development. Indonesia has recently mandated compulsory death registration across the entire country in December 2006. This article describes the methods and results from activities to ascertain causes of registered deaths in two pilot registration areas in Central Java during 2006-2007. The methods involved several steps, starting with adaptation of international standards for reporting causes of registered deaths for implementation in two sites, Surakarta (urban) and Pekalongan (rural). Causes for hospital deaths were certified by attending physicians. Verbal autopsies were used for home deaths. Underlying causes were coded using ICD-10. Completeness of registration was assessed in a sample of villages and urban wards by triangulating data from the health sector, the civil registration system, and an independent household survey. Finally, summary mortality indicators and cause of death rankings were developed for each site.

**Findings:**

A total of 10,038 deaths were registered in the two sites during 2006-2007; yielding annual crude death rates of 5.9 to 6.8 per 1000. Data completeness was higher in rural areas (72.5%) as compared to urban areas (52%). Adjusted life expectancies at birth were higher for both males and females in the urban population as compared to the rural population. Stroke, ischaemic heart disease and chronic respiratory disease are prominent causes in both populations. Other important causes are diabetes and cancer in urban areas; and tuberculosis and diarrhoeal diseases in rural areas.

**Conclusions:**

Non-communicable diseases cause a significant proportion of premature mortality in Central Java. Implementing cause of death reporting in conjunction with death registration appears feasible in Indonesia. Better collaboration between health and registration sectors is required to improve data quality. These are the first local mortality measures for health policy and monitoring in Indonesia. Strong demand for data from different stakeholders can stimulate further strengthening of mortality registration systems.

## Introduction

Measures of mortality are basic indicators for population health assessment, and are ideally derived using data from death registration systems. However, such data are not available for Indonesia,[1] the world's fourth largest population[2]. Indonesia has experienced steady socio-economic development over the past five decades, and has undergone demographic and epidemiologic transition resulting in important changes to population size, structure and health profile[3]. Timely and accurate information on mortality and causes of death is necessary to understand the direction and pace of these changes. However, civil registration in Indonesia has not yet realised its utility as a reliable data source for mortality measurement[4].

Under these circumstances, intermittent household surveys have been the principal source for mortality assessment in Indonesia[5]. (see Additional File 1) Available evidence on adult mortality and causes of death from surveys in Indonesia is limited on account of incomplete reporting of deaths[6]. Hence, indirect demographic methods and model life tables have been used to estimate mortality in Indonesia[6,7]. Also, as part of the Global Burden of Disease Study, the World Health Organization estimated mortality by age, sex and cause in Indonesia in 2001[8]. Total mortality was estimated using the WHO Model Life Table System,[9] and cause of death patterns for Indonesia were assumed to approximate a combination of patterns experienced in Singapore, Thailand, India and the Philippines[10]. Such estimates are not useful for regular monitoring of mortality levels and trends in Indonesia.

The Indonesian Ministry of Health has identified key measures of childhood and maternal mortality, and life expectancy at birth as indicators to chart health development in its 'Healthy Indonesia 2010' Plan[11]. These indicators are required at national, provincial and district level, as well as for urban centres. There is also a need to measure mortality from tuberculosis and HIV/AIDS for the United Nations Millennium Development Goals (MDGs)[12]. Also, In 2003-2004, an assessment of district health system performance in Indonesia identified a critical lack of information on mortality[13].

Therefore, the compilation of such data became a priority for the health sector. The Indonesian Mortality Registration System Strengthening Project (IMRSSP) was launched in 2006, towards developing a routine data source. The National Institute of Health Research and Development (NIHRD) coordinated IMRSSP activities in Indonesia, with technical assistance from the School of Population Health, University of Queensland. The broad goals of IMRSSP are to improve reporting of deaths and causes of death from health facilities and community based health centres, based on international standards. The initial objectives were to develop, test and implement cause of death reporting mechanisms in two pilot sites in Central Java. This paper reports the methods and processes developed and implemented in the IMRSSP, as well as an analysis of data collected in the two sites during 2006-2007.

## Methods

The urban municipality of Surakarta and the predominantly rural district of Pekalongan were chosen as the pilot sites for the project. The actual field implementation was preceded by a preparatory phase that involved several steps. At first, an inter-sectoral working group was constituted, which included representatives from NIHRD, the administrative and health sectors at central, provincial and district levels, and academic representatives. The working group first reviewed existing death registration procedures, and subsequently proposed a streamlined mechanism for death notification and registration at local level.(see Additional File 2) These procedures also incorporated international norms for reporting the cause of death, and compilation of mortality statistics[14]. The operational plans were then disseminated to the two pilot project areas for orientation of field staff. Extensive training programs were conducted for field interviewers, medical certifiers, and coders. This capacity building included in-class training sessions supplemented by instructional manuals and operational guidelines, [15-18] as well as on-site supervision and quality control throughout the implementation of IMRSSP.

Following a six month preparatory phase, data collection under IMRSSP was launched in January 2006 in the two sites. Baseline population data by age and sex for each area were obtained from the local offices of the Badan Pusat Statistik (Statistics Indonesia). For deaths in hospitals, attending physicians issued a death notification form to relatives, and also completed standard medical certificates of cause of death. For deaths outside hospitals, local health centre personnel first issued a death notification form to relatives, and later conducted household interviews to ascertain more details on the cause, using an Indonesian adaptation of draft versions of international standard verbal autopsy (VA) questionnaires[19]. Completed questionnaires were reviewed by trained health centre physicians who applied specific guidelines to attribute causes of death from VA [17,19]. All medical certificates of cause of death from hospitals and VA based death certificates from health centres are submitted to the district health office. Over here, trained coders apply rules to select and code the underlying cause for each hospital or home death, and these records are entered in the IMRSSP electronic database for subsequent analysis.

Completeness of data capture by IMRSSP was assessed in a sample of villages and urban wards in each site, through triangulation of data on deaths from different sources. In addition to the health sector data (i.e. IMRSSP), information on deaths during 2007 was obtained from two additional sources; the civil registration system; and deaths identified during an independent household survey conducted in December 2007. Data were matched across the three sources, using variables such as the name(s), age at death, gender, address and date of death. Allowances were made for matching the month rather than the exact date of death, and allowing a five year margin on the reported age at death from different sources, if other variables were matched (see Additional File 3 for details). Completeness of IMRSSP data was assessed as the proportion of IMRSSP deaths out of the total list of unique deaths derived from triangulation.

Life tables for males and females were developed for each study population site from the observed age-specific death rates. In addition, life tables were also computed after adjusting observed death rates using the measures of completeness described above. (details in Additional File 3) Underlying causes of reported deaths were aggregated by age and sex according to the ICD Mortality Tabulation List 1 [20] for primary tabulations of leading causes of death. Also, observed cause-specific mortality rates were standardised by age using the WHO international population standard, [21] and compared with similar rates reported for Indonesia in the Global Burden of Disease 2004 project[22].

## Findings

The contrasting socio-economic profiles and access to health care between urban Surakarta and rural Pekalongan serve as background information to study mortality differentials between the two sites. For indicators such as urbanization, literacy rates, and access to water supply and sanitation, Pekalongan approximates the national average for Indonesia. (see Table 1). Analysis of the population age structure indicated that Surakarta has a relatively older population than Pekalongan. Overall, data collection covered a total of 123 registration units and was accomplished through the services of 36 health facilities; which indicates the administrative and management support required for IMRSSP implementation.

**Table 1:** Summary of population and socioeconomic indicators for IMRSSP field sites and Indonesia in 2007

Parameter	Pekalongan	Solo	Indonesia
Population ^a^	263,397	557,544	218,868,791
Urban (% of population)	44.5	100.0	43.1
% pop ≥ 65 years	4.9	7.4	5.1
Human Development Index ^b^	68.2	76.0	69.6
Completed junior high school (% pop age 5+) ^b^	23.4	59.9	35.0
Private sanitary facilities (% of households) ^b^	47.1	75.7	58.5
Protected water source (% of households) ^b^	74.0	90.2	66.6
Number of villages/urban wards	72	51	-
Number of puskesmas ^c^	7	15	-
Number of hospitals	3	11	-

^a ^Data from BPS (Statistics Indonesia)

^b ^For Pekalongan, data are for all sub-districts in Kabupaten Pekalongan.

^c ^Puskesmas is an abbreviation for '*Pusat Kesahatan Masyakarat'*[Community Health Centre]

Data source [33]

**Figure 1:**
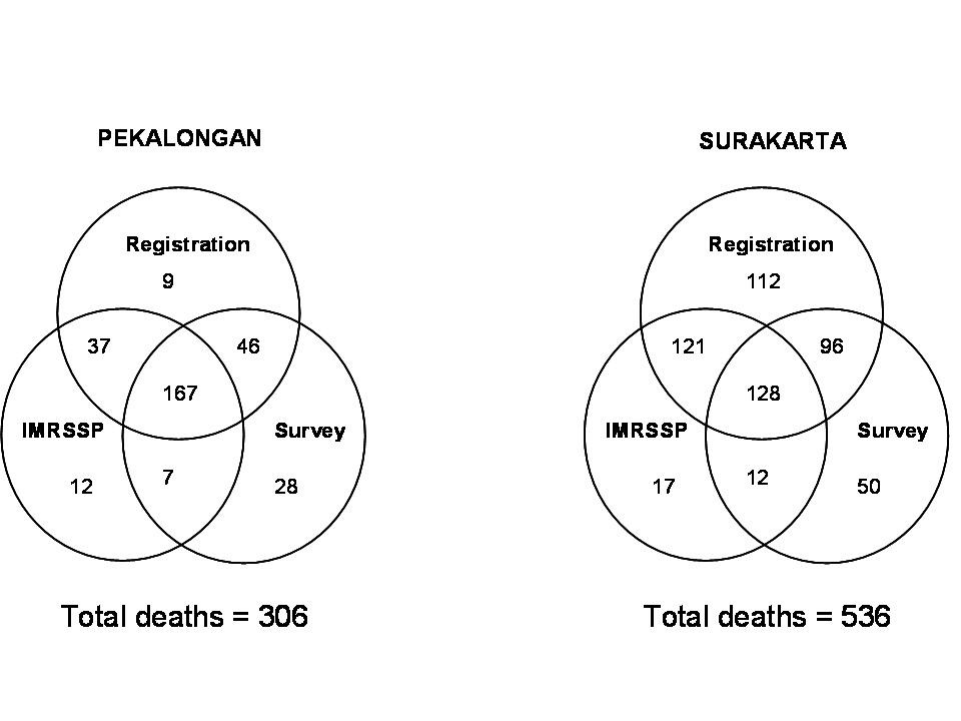
**Distribution of deaths from different data sources in sample populations in Pekalongan and Surakarta, to measure IMRSSP data completeness, 2007**.

The assessment of completeness of IMRSSP data in a sample of units demonstrated a marked variability in recording of information of deaths by the three different data sources. Figure 1 shows the distribution of deaths from the three sources used to compile the complete list of deaths from the sample units. The figure indicates that registration data from the *desa/kelurahan *(See Additional File 2 for definitions) offices has the highest proportion of completeness (> 85%), and also that combining data from registration and IMRSSP would result in over 90% completeness.

The summary mortality indicators in Table 2 indicate a three year differential between adjusted male and female life expectancy at birth in each site. Although there are only marginal differences in the risks of dying before age 5 years between the two sites, adult mortality rates are considerably higher in Pekalongan. This differential results in lower life expectancy at birth for both males and females in rural Pekalongan as compared to urban Surakarta.

**Table 2:** Reported and adjusted mortality measures for Surakarta and Pekalongan, 2006-2007

Site	Mortality indicator	Males		Females	
		**Reported**	**Adjusted **^**a**^	**Reported**	**Adjusted **^**a**^

**Surakarta**	Total deaths ^b^	3147	-	3337	-
	Crude death rate per 1000	5.8	-	5.9	-
	Estimated completeness	50.6%	-	53.1%	-
	Life expectancy at birth	75.7	63.5	79.5	66.2
	Under-five mortality ^c^	21.5	40.9	20.5	38.6
	Adult mortality rate ^d^	145	260	118	216
**Pekalongan**	Total deaths	1797	-	1754	-
	Crude death rate per 1000	6.8	-	6.7	-
	Estimated completeness	73.6%	-	72.1%	-
	Life expectancy at birth	68.1	62.8	70.8	65.6
	Under-five mortality	18.9	34.8	17.2	31.7
	Adult mortality rate	224	297	156	209

^a ^Mortality indicators calculated after adjusting death rates using age-specific measures of completeness of IMRSSP data (see Table 1 in Additional File 3)

^b ^Approximately 35% of deaths in Surakarta and 9% of deaths in Pekalongan were medically certified in hospitals

^c ^risk of dying between birth and exact age 5 years per 1000 live births;

^d ^risk of dying between exact ages 15 and 60 years per 1000 population

Table 3 compares the proportionate mortality distributions by cause between the two sites. While stroke is the leading cause, the presence of ischaemic heart disease and chronic obstructive lung disease among the leading causes is probably a reflection of lifestyle factors and environmental exposures that are common to both areas. However, the presence of tuberculosis and diarrhoeal diseases among leading causes of death in Pekalongan highlights the importance of infectious diseases in rural areas of central Java.

**Table 3:** Leading causes of death in Surakarta and Pekalongan, 2006-2007

ICD-10 codes	Surakarta		Pekalongan	
	
	Cause	%	Cause	%
I60-I69	Stroke	27.0	Stroke	19.9
E10-E14	Diabetes	8.5	Other heart diseases	7.5
I20-I25	Ischaemic heart disease	7.0	Chronic respiratory disease	7.1
I10-I14	Hypertensive diseases	6.4	Tuberculosis (A15-A19)	5.9
I26-I51	Other heart diseases	6.2	Ischaemic heart disease	5.9
C00-C97	Cancers	5.5	Hypertensive diseases	5.9
J40-J47	Chronic respiratory disease	4.4	Injuries	4.7
K70-K76	Liver diseases	4.4	Diarrhoeal diseases (A09)	4.6
V01-Y89	Injuries	3.7	Liver diseases	3.7
N00-N98	Renal diseases	2.8	Renal diseases	3.4
All other codes	All other causes ^a^	24.1	All other causes ^b^	31.3

^a ^Includes 7.3% of deaths from ill-defined causes

^b ^Includes 5.4% of deaths from ill-defined causes

**Figure 2:**
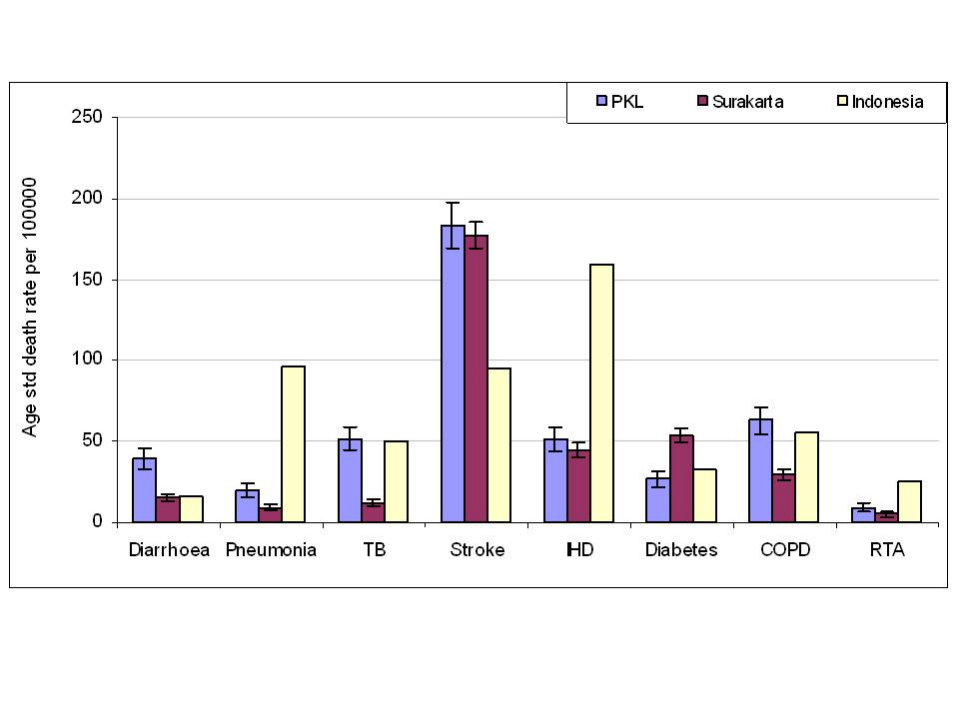
**Age standardised death rates (per 100,000) by cause for Pekalongan and Surakarta in 2006-2007, compared to Global Burden of Disease study estimates for Indonesia in 2004**.

Comparisons between observed age-standardised death rates per 100,000 population by cause (for males and females combined) from each field site with estimated rates for Indonesia reported in by the World Health Organization's Global Burden of Disease (GBD) study in 2004 [22] are presented in Figure 2. In addition to the major differences between the observed death rates and the WHO modelled estimates, particularly for stroke and ischaemic heart disease; the observed rates highlight the important sub-national differentials in cause-specific mortality from tuberculosis, diabetes, and chronic obstructive lung disease.

## Discussion

From a policy perspective, the initial findings presented for the two sites clearly indicate that in addition to the Millennium Development Goals, non-communicable diseases are also health priorities for Central Java. The adjusted under-five mortality rates (34-40 per 1000 live births) are at the upper end of the range reported by the Indonesian Demography and Health Survey program (32; 95% CI 21, 42) for Central Java for the period 1997-2007 [23]. For adults, the cause-specific mortality patterns highlight the magnitude of mortality from stroke and ischaemic heart disease in both urban and rural populations, and their contrast to the GBD estimates for Indonesia. The observed proportionate mortality from stroke in both sites is higher than reported (15%) by a recent national sample survey conducted in 2007, which also collected data on causes of death using VA methods[24]. These findings should lead to further epidemiological investigation of potential nutritional or behavioural determinants of cardiovascular disease in Indonesia, as conducted elsewhere[25]. Similarly, appropriate primary and secondary prevention strategies are required for diabetes, at least in urban areas. Also, cancer mortality needs more attention, in view of what is known on smoking trends in Indonesia,[26] and the current absence of population cancer registries in Indonesia[27]. The data also indicate the persistent burden from infectious diseases in the rural population. These findings could also be useful for future regional and global burden of disease estimates, and for comparative risk factor assessment.

While these findings represent the first local evidence on the estimated levels of mortality and causes of death within Indonesia, there are some important data limitations, including the completeness of IMRSSP data, and the validity of information on causes of death.

Incomplete data from both sites (particularly Surakarta) is a matter of concern (see Additional file 3), despite the reported crude death rates of 5.8 - 6.8 per 1000 for the two sites being much higher than the crude death rate of 3.3 per 1000 reported from a national survey in 2007[28]. The completeness of data could be improved by strengthening the registration guidelines to require both *de facto *and *de jure *registration. There is also the need to sensitise all stakeholders about the need for reporting deaths at the extremes of age. The most important issue would however, be the need to strengthen intersectoral collaboration, given that over 90% of deaths are likely to be recorded either by the administrative or the health sector. (See Additional file 3) Such collaboration can be achieved by the health system being proactive in periodically obtaining lists of deaths recorded by the local administration. Also, occasional coordination sessions could be conducted to orient registration and health sector personnel about their roles in IMRSSP. Based on the findings from this research, the Government of Indonesia has recently published a joint regulation from the Ministries of Home Affairs and Health, authorising collaborative efforts between offices from each ministry at all levels, to strengthen mortality and cause of death registration in Indonesia[29].

Another potential limitation is the validity of reported causes of death. About 37% of deaths in Surakarta and 9% in Pekalongan are medically certified in hospitals, and it would be reasonable to assume accurate reporting of the cause for these deaths. However, research elsewhere has identified problems even in medically certified deaths, although the observed misclassification patterns were compensatory[30,31]. On the other hand, the challenges in attributing causes of death using VA methods are well known[32]. Hence, continued implementation of IMRSSP should also include specific efforts to measure the reliability and where possible, validity of reported causes, both from VA and medical certification. Also, forensic and/or police data systems should be explored to identify deaths from injuries that are missed by the current IMRSSP data collection processes, leading to regular data sharing between these systems. Similarly, information should also be shared between registration and other health programs that have their own case and mortality notification systems (e.g. maternal and child health; tuberculosis control). The results from such activities would improve data completeness, and enable appropriate interpretation and therefore utility of cause-specific mortality data from the project.

This project has developed a model to strengthen death registration systems as a source of empirical data to measure total and cause-specific mortality in Indonesia. From an operational perspective, the health sector was targeted for strengthening mortality notification. This approach was adopted since deaths either occur in health facilities, or are brought to the notice of community health personnel in the course of their functions, or, as per the recent legislation, documentary evidence of death is required from health personnel. Also, the processes for collection, compilation and analyses of data on age, sex and cause-specific mortality require health personnel with specific training, which was provided in this project. Nevertheless, these functions are best served when integrated with the legal and administrative processes for vital registration.

**Figure 3:**
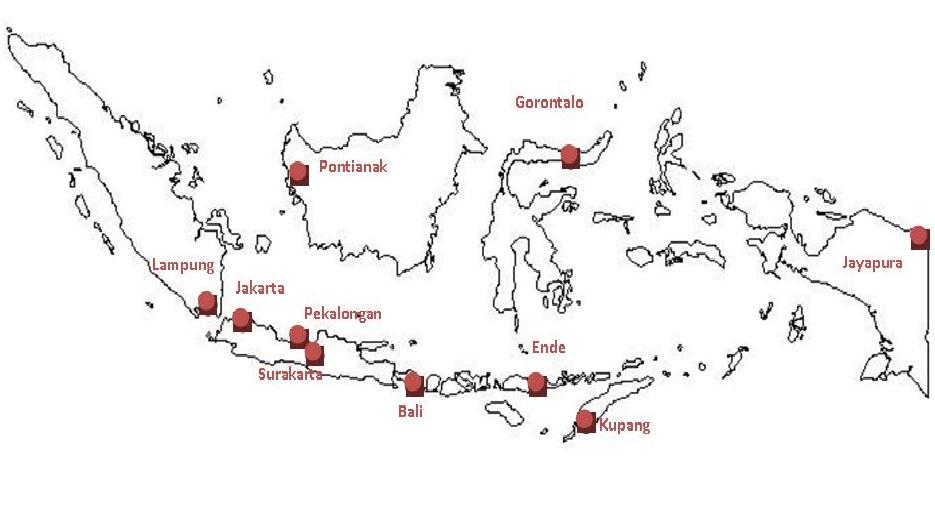
**Distribution of IMRSSP field sites in Indonesia in 2009-2010**.

Hence, in recognition of the systematic approach adopted in the IMRSSP, and the urgent need for local evidence on mortality across Indonesia, the project has been expanded to sites located on four other major islands of Indonesia; Sumatra, Kalimantan, Sulawesi, and Papua in 2007-2008; and to sites in Bali and Nusa Tenggara Timur (NTT) in 2009. (see Figure 3).

## Conclusions

Implementing cause of death reporting in conjunction with death registration appears feasible in Indonesia Improved inter-sectoral collaboration between registration and health departments, along with a role for local offices of Statistics Indonesia (BPS) in the compilation, processing and submission of vital statistics would increase the availability of mortality data at the district and provincial levels. Continued implementation of IMRSSP over the next 5 to 10 years, supported by appropriate operational research activities to measure and improve data quality is necessary to develop a routine source of mortality data for health policy and monitoring in Indonesia.

## Competing interests

The authors declare that they have no competing interests.

## Authors' contributions

CR and SS conceptualised the design of IMRSSP, contributed to data analysis and interpretation. CR drafted the initial version of the manuscript. CR, SS, SD, S, YW, LP and SK participated in the data collection, analysis and interpretation. TA and JI facilitated data management and quality control, and participated in data analysis and interpretation. ADL contributed to data interpretation. All co-authors contributed to revisions and preparation of the final version of the manuscript.

## Supplementary Material

Additional file 1 **Data sources for estimating mortality in Indonesia, 1971-2007**. Click here for file

Additional file 2 **Death registration procedures in Indonesia**. Click here for file

Additional file 3 **Estimating data completeness**. Click here for file
