# Supplementary material for: Mortality in Central Java: results from the indonesian mortality registration system strengthening project
Source: BMC Res Notes. 2010 Dec 2;3:325. doi: 10.1186/1756-0500-3-325 (PMC3016265; doi:10.1186/1756-0500-3-325)
Supplement: Additional file 1 — Data sources for estimating mortality in Indonesia, 1971-2007. [file 1756-0500-3-325-S1.PDF]

Additional File 1: Data sources for estimating mortality indicators in Indonesia, 1971-2007

**Data source**	**Data availability (enumeration years)**		
	**Child mortality** [a]	**Adult mortality** [b]	**Causes of death** [c]
Demographic and Health Survey (DHS)	87, 91, 94, 97, 03, 07	94, 97, 03, 07	-
Population Census	71, 80, 90, 00	00	-
Inter-Censal Survey (SUPAS)	76, 85, 95, 05	85	-
National Socio-Economic Survey (SUSENAS) [d]	80, 86, 92, 95, 01, 07	80, 86, 92, 95, 01, 07	80, 86, 92, 95, 01, 07
Baseline Health Survey RISKESDAS)	07	07	07

[a] DHS provides direct child mortality estimates based on detailed birth histories, all other sources produce indirect estimates based on the 'children ever borne and surviving' method

[b] DHS provides adult mortality estimates based on sibling survival methods; all other sources provide estimates based on direct household recall of deaths during specified reference periods

[c] Based on verbal autopsy methods

[d] The SUSENAS is conducted annually, but the population module is included on an intermittent basis
