# Supplementary material for: Mortality in Central Java: results from the indonesian mortality registration system strengthening project
Source: BMC Res Notes. 2010 Dec 2;3:325. doi: 10.1186/1756-0500-3-325 (PMC3016265; doi:10.1186/1756-0500-3-325)
Supplement: Additional file 2 — Death registration procedures in Indonesia. [file 1756-0500-3-325-S2.PDF]

Additional File 2

Death registration procedures in Indonesia

Civil registration (including births and deaths) was first instituted in Indonesia under Dutch colonial government regulations (*Staatblaad*) in 1847; subsequently revised in 1929, 1933 and 1946 (cited in [1]). Under these regulations, the implementation of death registration had been limited largely to urban areas, covering less than 25% of the national population.[1] A review of available data in 2001 indicated that vital statistics were found to be of low completeness, reliability and validity.[2] Also, requirements for generating evidence for civil registration, corpse disposal and health information resulted in use of separate forms and reporting mechanisms to different authorities, leading to multiple, differing compilations of mortality statistics in Jakarta.[3]

In 2006, the Indonesian government enacted a new law on Civil Administration which mandates compulsory birth and death registration throughout the country. [4] The office of the Directorate General of Population Administration within the Ministry of Home Affairs is entrusted with the charge of civil registration operations. Draft versions of the law, supported by provisional guidelines for its implementation [5] were tested in pilot projects in two areas of Central Java province in 2004; Surakarta (urban); and Pekalongan (a predominantly rural district). Registration is required within 30 days at place of occurrence, at the village administration office (*desa*) in rural areas, or the urban administration offices (*kelurahan*) in urban areas. Notification of deaths to the *desa* or *kelurahan* office is the citizen's responsibility, and should have supporting documentation from a doctor or paramedic as evidence of the event.[4]

In rural areas, local community volunteers (*rukun tettanga (RT) & rukun warga(RW)*) are expected to facilitate notification of deaths in the community to the local health centre *(puskesmas)* for supporting documentation, and then to the village headman (*kaur kesra)* to complete the procedures. In urban areas, relatives of the deceased submit the a document provided by the hospital where death occurred, or for home deaths, the family approaches the local health centre for the required document. At the *desa* or *kelurahan* office, a death record (*surat ketarangan kematian (SKK)*) is issued, which is taken to the sub district or district administration to obtain an official death certificate for legal and social purposes. The law also requires the compilation of vital statistics at district, provincial and national level, through the Information System for Civil Administration.

Although the law does not specify the need to report the cause of death, existing formats used by health authorities include space to state the cause. However, our initial review has identified wide variations in the design and structure of these formats across different locations in Indonesia. Based on the stated cause(s), the registrar records the cause in the SKK using one of the following six categories: general disease/sickness in elderly; plague; accident; crime; suicide; or other reason to be specified.[5] This does not conform to international standards for reporting causes of death at death registration.[6]

The Indonesian Mortality Registration System Strengthening Project (IMRSSP) has been designed to improve the completeness of death registration as well as institute formal mechanisms for recording the cause of death at registration, using medical certification for deaths in health facilities, and verbal autopsy methods for deaths at home. The project has developed new instruments for recording the cause of death, operational manuals and guidelines for field operations, and has conducted extensive

capacity building activities. The project has also facilitated the development of government regulations to support implementation. The Project is being implemented through the offices of the Ministry of Health, and is being coordinated by the National Institute of Health Research and Development (NIHRD). Based on initial findings of IMRSSP, close collaboration is being sought between the health sector and local offices of population administration at the village, district and provincial levels, towards improving the quality of registration data for public health and epidemiology in Indonesia.

References

1. Hartono B: **Status of mortality statistics in Indonesia**. In *Regional Consultation on Mortality Statistics: 2007; New Delhi*. WHO Regional Office for South East Asia:SEA-HSD-304; 2007.
2. Oye-Gardiner M, Gardiner P: **Reform of Citizen Administration in Indonesia:**. In *Results of the National Conference on Improving Public Services in Citizen Administration, organized by Kementerian Pendayagunaan Aparatur Negara, and Departemen Dalam Negeri, Republic of Indonesia: 2002; Jakarta, RI*; 2002.
3. Rajagukguk O: **Death Rate and Rapid Assessment on situation in DKI, Jakarta**. Jakarta: Demographic Institute, Economic Faculty of Indonesia University; 2003.
4. Government of Indonesia: **Undang-Undang Republik Indonesia Nomor 23 Tahun 2006 tentang Administrasi Kependudukan *[Indonesian Republic Law 23/2006 about Population Administration*]**. *Volume 23/2006*. Government of Indonesia,Jakarta; 2006.
5. DGPA: **Panduan: Pencetatan Kelahiran, Lahir Mati dan Kematian [*Guidelines on the reporting of births, stillbirths and deaths*].** Jakarta: Direktorat Jenderal Administrasi Kependudukan,Departemen Dalam Negeri [*Directorate General of Population Administration (DGPA), Ministry of Home Affairs*] Indonesia 2003.
6. WHO: **Mortality: guidelines for certification and rules for coding**. In *International Statistical Classification of Diseases and Health Related Problems - Tenth Revision Volume 2: Instruction Manual. Volume 2*. Geneva: World Health Organization; 1993:30-65.
