# Supplementary material for: Mortality in Central Java: results from the indonesian mortality registration system strengthening project
Source: BMC Res Notes. 2010 Dec 2;3:325. doi: 10.1186/1756-0500-3-325 (PMC3016265; doi:10.1186/1756-0500-3-325)
Supplement: Additional file 3 — Estimating data completeness. [file 1756-0500-3-325-S3.PDF]

Additional File 3

Estimating data completeness

To assess completeness of IMRSSP data, an independent household survey was conducted in a representative sample of villages in December 2007, by staff from the district office of the Badan Pusat Statistik Indonesia (Statistics Indonesia). The survey covered 10,467 households in 12 *desa* (villages) in Pekalongan; and 14,607 households in 13 *kelurahan* (urban ward) in Surakarta. The survey was conducted in conjunction with a household register update activity for electoral purposes. Each household in the sampled villages was asked to report deaths that had occurred during the survey reference period (January-October 2007). For each village, lists of deaths during the reference period were available from three sources:

a) The household survey described above
b) IMRSSP data for each *desa/kelurahan*; as compiled from the deaths notified by hospitals or health centres (see Methods in main manuscript)
c) Death registration data from the sub-district population administration office – which receives routine monthly returns on registered deaths from the head of the *desa/kelurahan*

Data was matched across the three sources. The variables used for matching included the name(s), age at death, gender, address, and date of death. All these variables were used by the research team to adjudicate on matching, making allowances for matching on the month rather than the exact date of death, and allowing a five year margin on the reported age at death from different sources, if other variables were matched. Once the matching process was completed, a list of unique deaths that were reported from at least one of the three data sources was compiled for each village, and aggregated for all sampled units in each of the two field sites. These lists were

considered to be the potential complete lists of deaths during the reference period for these sample units. Figure 1 below shows the distribution of deaths from the three sources used to compile this complete list of deaths in each site.

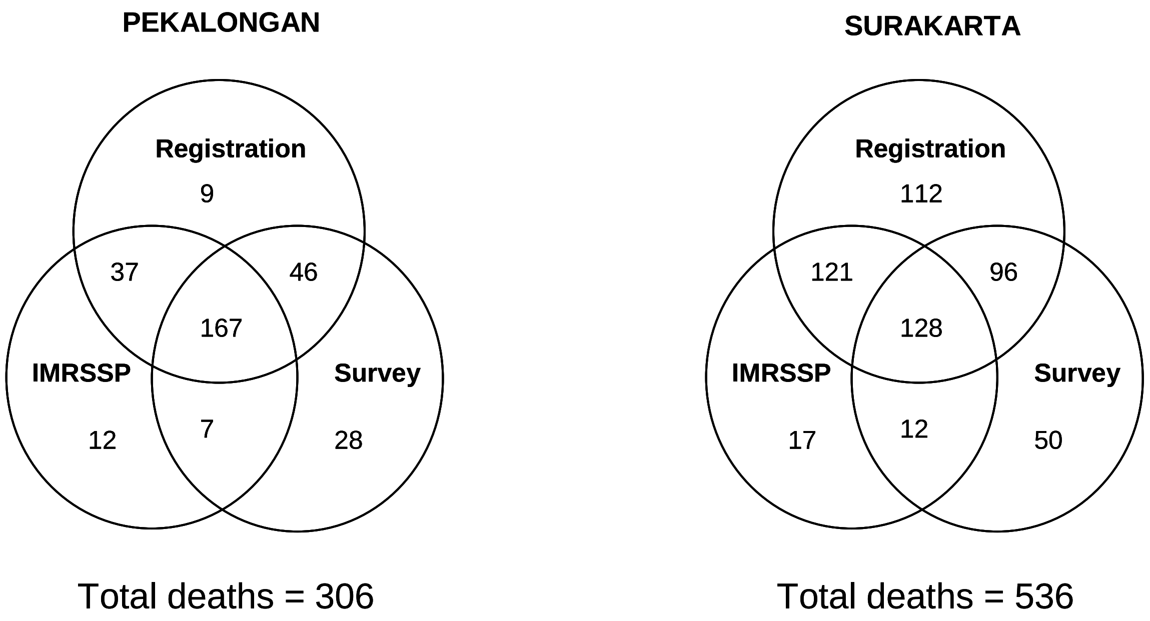

Figure 1: Distribution of deaths by data source from sample areas in each field site, 2007

The data represented in Figure 1 indicates the variability in recording of information of deaths in the three different data sources. We chose to estimate the completeness of each data source as the proportion of deaths recorded out of the total deaths from all three sources. Completeness was estimated by sex and three broad age groups, 0-14 years, 15-59 years, and >60 years. (see table below).

The results indicate limitations in the completeness of IMRSSP data from both sites, and identified that overall, IMRSSP recorded only about 73% of deaths in Pekalongan, and 52% of deaths in Surakarta. There were minimal differences in completeness for males and females, and in both locations, completeness of IMRSSP data was highest at ages above 60 years. These estimates of completeness were used

to adjust reported age-sex specific death rates from the overall IMRSSP data from each site, and the adjusted rates were used to construct life tables and report summary mortality measures in Table 2 in the main manuscript.

Table 1: Percentage of deaths recorded by IMRSSP out of total deaths from all sources in the representative sample of villages from Pekalongan and Surakarta, 2007

Population variable	Pekalongan		Surakarta	
	Deaths from all sources	Deaths in IMRSSP data	Deaths from all sources	Deaths in IMRSSP data
**Gender**				
Male	163	73.6%	259	50.6%
Female	143	72.0%	277	53.1%
**Age**				
0-14	28	53.8%	28	53.8%
15-59	88	72.1%	140	40.6%
60+	190	75.7%	368	55.7%

Interpretation of findings

A significant aspect from the data completeness assessment is that the registration data from the *desa/kelurahan* offices have the highest completeness, capturing about 85% of the complete list of deaths in both sites. On combining the data from registration with the data from health facilities, it is noted that these two sources cover about 91% of the total deaths; and only about 9% of additional deaths were identified the household survey. Examination of the data also revealed that the survey too missed a number of deaths that were recorded in either of the two routine sources, particularly in Surakarta. The implications of these findings are that adequate collaboration between the two routine sources is likely to yield high levels of

completeness of data, and will reduce the need to mount independent surveys which are costly, and the data collected from which are subject to information or recall bias.

These completeness estimates could be influenced by the criteria applied to match events from different data sources. The *desa/kelurahan* offices as well as health centres often record only one name, or only the household name of the deceased, creating difficulties in matching events. Also, our observations during the independent household survey indicated that there was more emphasis on household enumeration, with less attention being paid to the recording of deaths. Therefore, for our analyses, we chose to use triangulation of data across the three sources, rather than use formal 'capture-recapture' methods to estimate the completeness of death recording from each source.[1] In our analyses, we applied strict criteria to consider any potential mismatches by name as unique events, rather than reconcile the data based on assumptions, and hence may have double counted rather than missed deaths in our final list. This could have led to an under estimation of completeness of IMRSSP data.

On the other hand, qualitative research on community perceptions of death registration has indentified that there are instances of deaths among chronically ill elders, as well as stillbirths and infant deaths that are not notified to either administrative or health authorities.[2] Also, some deaths of usual residents which occur outside the study areas (e.g. in tertiary care facilities in Jakarta) are not notified locally, since notification is required only at place of occurrence. Hence, IMRSSP data is likely to be incomplete, and we chose to adjust the reported mortality rates using the measures of completeness based on triangulation. Therefore, our study may

have yielded potentially lower completeness estimates of IMRSSP data, leading to larger adjustments to the summary mortality indicators.

In conclusion, this study to estimate the completeness of registration indicates that close collaboration between health and administration sectors could improve the completeness of death registration in Indonesia. However, our qualitative research also indicated that such collaboration at the local level can be effected only through formal government authority. As a consequence of this research, the Government of Indonesia has published a joint regulation from the Ministries of Home Affairs and Health, authorising collaborative efforts between offices from each ministry at all levels, to strengthen mortality and cause of death registration in Indonesia. [3]. Similar agreements are being published at the provincial, district and municipality level in all field sites where IMRSSP is currently being implemented. We believe that such regulations would be instrumental for improved completeness and quality of Indonesian mortality data into the future.

References

1. Chandrasekar C, Deming W: **On a method of estimating birth and death rates and the extent of registration**. *Journal of American Statistics Association* 1949, **44**:101.
2. Jennaway M, Rachmalina I, Rao C, Kosen S, Hill P: **Culturally sensitive methods of eliciting information on cause of death (verbal autopsies) in local communities: Indonesian case studies. A report to the Health Metrics Network, World Health Organization, Geneva.** Brisbane: University of Queensland, Australia & National Institute of Health Research and Development, Indonesia.; 2009.
3. **Menteri Dalam Negeri dan Menteri Kesehatan [*Ministry of Home Affairs and Ministry of Health*], 2010.** Peraturan bersama pelaporan kematian dan penyaban kematian *[Joint regulation on reporting of mortality and causes of death*], Government of Indonesia. Nomor 15 Tahun 2010; nomor 162/MENKES/PB/I/2010.
